# Pollen data from bottom sediments of a tundra lake in the Yerkuta River basin on the Yamal Peninsula

**DOI:** 10.1016/j.dib.2022.108539

**Published:** 2022-08-19

**Authors:** Gulnara Nigamatzyanova, Niyaz Nigmatullin, Oleg Tumanov, Bulat Gareev, Larisa Frolova

**Affiliations:** Kazan (Volga Region) Federal University^1^

**Keywords:** Arctic, Pollen, Spore, Vegetation history, Holocene

## Abstract

This article extends the findings of our previous research “Preliminary reconstruction of climate changes and vegetation cover inferred from pollen study of the arctic lake bottom sediments from the southwestern part of the Yamal Peninsula” (G.R. Nigamatzyanova, N.M. Nigmatullin, B.I. Gareev, O.N. Tumanov, L.A. Frolova, 21st International Multidisciplinary Scientific GeoConference SGEM. 4 (2021) 415-421. https://doi.org/10.5593/sgem2021/4.1/s19.53) [1].

The Late Holocene vegetation history of the southern part of the Yamal Peninsula was reconstructed using the pollen data obtained from bottom sediments of the tundra lake K1 (68°15.320′ N, 69°07.675′ E) near the Yerkuta research station in the Yerkuta River basin. A 30-cm-long sediment core was drilled during the expedition to the lake in 2014. A total of 15 samples were taken at 1-cm intervals for palynological analysis. To extract pollen grains, prior chemical treatment of the samples by the standard methods. The palynological dataset was exported in Excel sheets, one listing the raw pollen counts and the other containing the pollen percentages determined with respect to the total pollen counts for all taxa. The palynodiagram illustrating the variations in pollen and spore percentages with depth is given. These palynological records yield insight into the vegetation dynamics on the Yamal Peninsula in response to the climate forcing and may be of practical importance in regional syntheses of the vegetation history in the region.

**Specifications Table**

**Table:** 

Subject	Earth and Planetary Sciences
Specific subject area	Palynology, paleoecology, paleoclimate, Arctic, tundra lake, sediments, Holocene
Type of data	Table, figure
How the data were acquired	Equipment for field sampling: UWITEC gravity corer. Dried samples were treated with heated 10% HCl solution, 10% KOH solution, sieved through a 400 µm mesh and heated HF according to the Faegri and Iversen’ method. Residues were mounted on microscope slides using glycerol. Microscope analysis was carried out with a Carl Zeiss Axio Imager A2 bright field illumination and 400x magnification.
Data format	Analyzed
Description of data collection	Up to 330 pollen grains and spores were counted per sample. The calculation of pollen percentages is based on the total sum of pollen grains excluding spores. Calculation of the pollen concentration, construction of the palynological diagram have been performed using the Tilia/TiliaGraph software.
Data source location	Institute of Geology and Petroleum Technologies, Kazan (Volga Region) Federal University Kazan, Republic of Tatarstan Russia Coring location 68°15.320′ N, 69°07.675′ E
Data accessibility	Repository name: PANGAEA DOI: https://doi.org/10.1594/PANGAEA.939401 Nigamatzyanova, Gulnara; Frolova, Larisa A (2021): Pollen and spore record of lake sediments, the southern part of the Yamal peninsula, Russia. PANGAEA Repository name: Mendeley Data Direct URL to data: DOI: 10.17632/yspzsj4c63.1 Nigamatzyanova Gulnara; Frolova Larisa (2022), “Palynomorphs of Holocene lake deposits of the Yerkuta River Basin (the Yamal Peninsula)”, Mendeley Data, V1. License CC BY 4.0
Related research article	G.R. Nigamatzyanova, N.M. Nigmatullin, B.I. Gareev, O.N. Tumanov, L.A. Frolova Preliminary reconstruction of climate changes and vegetation cover inferred from pollen study of the arctic lake bottom sediments from the southwestern part of the Yamal Peninsula. 21st International Multidisciplinary Scientific GeoConference SGEM. 4 (2021) 415-421. https://doi.org/10.5593/sgem2021/4.1/s19.53

## Value of the Data

• Since lake K1 is the one that has been previously unexplored in the Yerkuta River basin, it is of particular interest to look into the changes that its ecosystem underwent in the past. The sediment core under study provides a continuous palynological record for this tundra lake during the Late Holocene. The stratigraphic distribution of the palynomorphs retrieved appears to be indicative of the environmental conditions of that time.
• The results of this study contribute significantly to future research on environmental dynamics, climate change, human impacts on the environment, and vegetation responses to both environmental and climatic factors. These data can be an aid to palynologists, paleoclimatologists, and paleobotanists. The dataset obtained can be a helpful resource for regional syntheses of the vegetation dynamics in the Yamal Peninsula [1,2]. The pollen records can be merged with any other similar datasets to develop a large database on the relationship between the proxy indicators (such as cladocerans, diatoms, etc.) [3], [4], [6] and paleoenvironmental conditions of the past.

## 1 Data Description

The palynological dataset was exported in Excel sheets, one listing the raw pollen counts (Table 1) and the other containing the pollen percentages (Table 2) determined with respect to the total pollen counts for all taxa.

**Table 1:** Pollen counts of the identified taxa from the bottom sediments of lake K1.

**Table 2:** Pollen percentages of the identified taxa from the bottom sediments of lake K1.

**Fig. 1:**
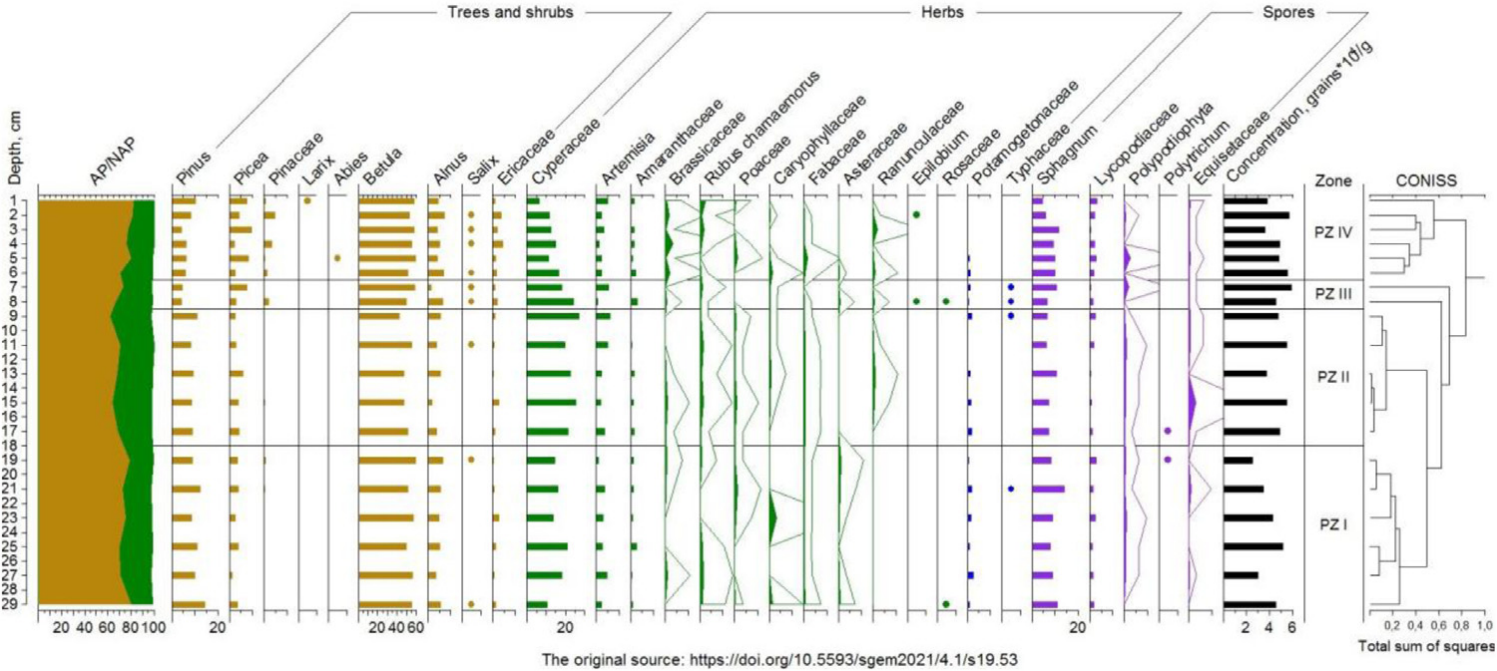
Palynodiagram showing the percentages of pollen grains and spores of the identified dominant taxa relative to the depth of each section of the sediment core taken from lake K1.

The palynodiagram illustrating the variations in pollen and spore percentages with depth is given in Fig. 1. The total pollen sum of arboreal and non-arboreal plants was taken as 100%. The curves were exaggerated (up to 10 times). The dots show the qualitative occurrence. The AP/NAP values are the ratios between arboreal and non-arboreal taxa.

## 2 Experimental Design, Materials and Methods

The Late Holocene vegetation history of the southern part of the Yamal Peninsula was reconstructed using the pollen data obtained from bottom sediments of the tundra lake K1 (68°15.320′ N, 69°07.675′ E) near the Yerkuta research station in the Yerkuta River basin. The lake is small-sized (surface area 0.43 km^2^, maximum depth 6.5 m) and located within the shrub tundra subzone characterized by subarctic climatic conditions [6,7]. A 30-cm-long sediment core was drilled by the UWITEC gravity corer during the expedition to the lake in 2014. The core was divided into 30 samples using a core cutter. A total of 15 samples (2.03–2.06 g each) were taken at 1-cm intervals for palynological analysis. To extract pollen grains, prior chemical treatment of the samples by the standard methods was performed: the samples were treated with heated 10% HCl and then 10% KOH, sieved through a 400 µm mesh, and exposed to heated HF according to Faegri and Iversen's method (bypassing the acetolysis stage) [8]. Each sample was spiked with a tablet of Lycopodium clavatum spores (Lund University, Batch No. 1031, 20848 ± 691 spores per tablet) to measure the concentration of pollen grains [9]. The residues were mounted on microscope slides using glycerol for subsequent examination under a Carl Zeiss Axio Imager A2 microscope at 400x magnification in the bright-field imaging mode. Up to 330 pollen grains and spores were counted per sample. Identification of pollen and spores was carried out with the help of Kupriyanova and Aleshina's keys [10,11]. The pollen percentages were determined from the total pollen sum (excluding spores). The results were visualized as a spore-pollen diagram with the help of the Tilia/TiliaGraph software [12].

## Ethics Statements

The work did not involve the use of human subjects, animal experiments and data collected from social media platforms.

## CRediT authorship contribution statement

**Gulnara Nigamatzyanova:** Writing – original draft. **Niyaz Nigmatullin:** Methodology, Software. **Oleg Tumanov:** Writing – review & editing. **Bulat Gareev:** . **Larisa Frolova:** .

## Declaration of Competing Interest

The authors declare that they have no known competing financial interests or personal relationships that could have appeared to influence the work reported in this paper.

## Data Availability

Pollen and spore record of lake sediments, the southern part of the Yamal peninsula, Russia (Original data) (PANGAEA).
